# Dissolution–Precipitation Synthesis and Characterization of Zinc Whitlockite with Variable Metal Content

**DOI:** 10.1021/acsbiomaterials.1c00335

**Published:** 2021-07-28

**Authors:** Agne Kizalaite, Inga Grigoraviciute-Puroniene, Dane Romar C. Asuigui, Sarah L. Stoll, Sung Hun Cho, Tohru Sekino, Aivaras Kareiva, Aleksej Zarkov

**Affiliations:** † Institute of Chemistry, Vilnius University, Naugarduko 24, LT-03225 Vilnius, Lithuania; ‡ Department of Chemistry, Georgetown University, 37th and O Streets NW, Washington, D.C. 20057, United States; § SANKEN (The Institute of Scientific and Industrial Research), Osaka University, 8-1 Mihogaoka, Ibaraki, Osaka 567-0047, Japan

**Keywords:** zinc whitlockite, dissolution−precipitation, phase conversion, thermal stability

## Abstract

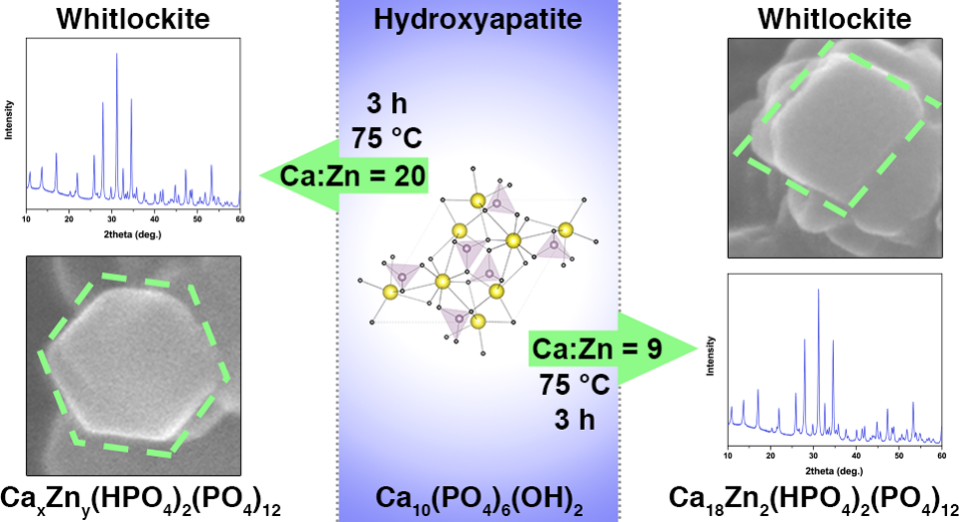

In the present work, a series of zinc whitlockite (Ca_*x*_Zn_*y*_(HPO_4_)_2_(PO_4_)_12_) powders was synthesized by a low-temperature dissolution–precipitation process for the first time. The phase conversion from calcium hydroxyapatite to zinc whitlockite occurred in an acidic medium in the presence of Zn^2+^ ions. Variable chemical composition of the synthesis products was achieved by changing Ca-to-Zn molar ratio in the reaction mixture. Investigation of the phase evolution as a function of time demonstrated that phase-pure zinc whitlockite powders can be synthesized in just 3 h. It is also demonstrated that single-phase products can be obtained when the Ca-to-Zn ratio in the reaction medium is in the range from 9 to 30. With higher or lower ratios, neighboring crystal phases such as scholzite or calcium hydroxyapatite were obtained. The morphology of the synthesized powders was found to be dependent on the chemical composition, transforming from hexagonal to rhombohedral plates with the increase of Zn content. Thermal stability studies revealed that the synthesized compounds were thermally unstable and decomposed upon heat treatment.

## 1 Introduction

Calcium phosphates (CPs) represent the most widespread class of ceramic biomaterials used for bone regeneration purposes due to their excellent biological performance and similarity in chemical composition to the natural bone.^1^ Despite the high biocompatibility of non-ion-substituted CPs, the partial substitution of calcium or phosphate ions is commonly employed for the preparation of CPs with improved biological properties. Synthetic CPs substituted with other biologically active ions can be considered as a sub-group of the CP family while possessing specific properties provided by incorporated foreign ions.^2,3^ This approach appears especially reasonable due to the fact that biological CPs contain significant amounts of other ions.^4^

Magnesium whitlockite [Mg-WH, Ca_18_Mg_2_(HPO_4_)_2_(PO_4_)_12_] can be considered as a Mg-substituted CP, which naturally occurs in humans. This compound is known to be the second most abundant biomineral in human hard tissues constituting around 20–35 wt % of the total inorganic components of the bone.^5^ The crystal structure of synthetic Mg-WH was described by Gopal et al.^6^ It was determined that WH crystals have a space group of *R*3*c* (#161) with hexagonal parameters *a* = 10.350(5) and *c* = 37.085(12) Å. Although structural relationship between WH and β-tricalcium phosphate [β-TCP, Ca_3_(PO_4_)_2_] has been resolved, these two names are often used interchangeably and synonymously. The reason is high similarity of the X-ray diffraction (XRD) patterns of both compounds, which makes it almost impossible to distinguish between these materials. Nevertheless, unlike WH, pristine β-TCP contains only Ca cations and neither β-TCP nor its Mg-substituted version contains HPO_4_^2–^.

Despite the presence of high content of Mg-WH in the human body, it is not so widely used in clinics, basically due to the challenges in the preparation of this material. Nevertheless, in recent years, Mg-WH attracted significantly more attention as a number of studies reported various synthetic approaches and characterization of Mg-WH.^7−13^ It was demonstrated that Mg-WH possesses some superior properties compared to those of frequently used biomaterials such as calcium hydroxyapatite [HAp, Ca_10_(PO_4_)_6_(OH)_2_] or TCP. The comparative study on in vitro and in vivo biocompatibility of Mg-WH, HAp, and β-TCP revealed that Mg-WH-containing scaffolds facilitated bone-specific differentiation in comparison with HAP-reinforced composite scaffolds. Moreover, WH implants induced comparable or even better bone regeneration in calvarial defects in a rat model compared to HAP and β-TCP implants.^14^ According to Kim et al.,^15^ under physiological conditions, Mg-WH nanoparticles can recapitulate the early stage of bone regeneration through stimulating osteogenic differentiation, prohibiting osteoclastic activity, and transforming into HAp-neo bone tissues. It was shown that the phase transformation from Mg-WH into HAP is a key factor leading to the rapid bone regeneration with a denser hierarchical structure. Comparison of HAp/chitosan and Mg-WH/chitosan scaffolds revealed that the Mg-WH/chitosan composite possessed better biocompatibility, enhancing proliferation and osteogenic differentiation ability of human mesenchymal stem cells. In addition, Mg-WH-containing scaffolds significantly promoted bone regeneration in calvarial defects.^16^

Despite the fact that the ionic radius of Mg^2+^ is very similar to those of the first-row divalent transition-metal (TM) ions,^17^ reports on the synthesis of TM-WH are almost absent. The rare example of TM-WH was published by Belik et al.,^18^ who prepared Ca_9_FeD(PO_4_)_7_ by treating Ca_9_Fe(PO_4_)_7_ with D_2_ at elevated temperatures. Earlier, Kostiner and Rea reported the crystal structure of accidently synthesized manganese WH (Mn-WH).^19^ To the best of our knowledge, there are no reports in the literature regarding the zinc analogue–zinc whitlockite (Zn-WH). At the same time, Zn is known to be an essential and biologically active ion, which is involved in many biological processes in the human body, and its’ deficiency leads to a number of skeletal anomalies.^20^ The Zn content in the human bone varies from 0.012 to 0.025 wt %, which is relatively high compared to other tissues.^21^ It should also be mentioned that Zn is a relatively harmless element; thus, many studies on synthetic Zn-substituted CPs report much higher levels of Zn doping with respect to physiological amounts.^22^ Different Zn-substituted CPs such as HAp, β-TCP, and monetite demonstrated superior biological performance; moreover, Zn ions are known for their antibacterial properties.^23−28^

In the present work, we report the low-temperature synthesis of Zn-WH by a time- and cost-effective dissolution–precipitation process. We believe that this material has strong potential to be used in applications where Mg-WH has proven to be superior in biocompatibility and bone regeneration but has so far proven to be challenging synthetically. Further, in addition to the simplicity of the synthesis, we have demonstrated a wide range of composition stabilities by successfully synthesizing and characterizing a series of phase-pure Zn-WH powders containing different amounts of Ca and Zn ions. The composition of the products could be achieved by changing the Ca-to-Zn ratio in the reaction mixture, leading to remarkable phase stability.

## 2 Materials and Methods

### 2.1 Synthesis

**Figure 1:**
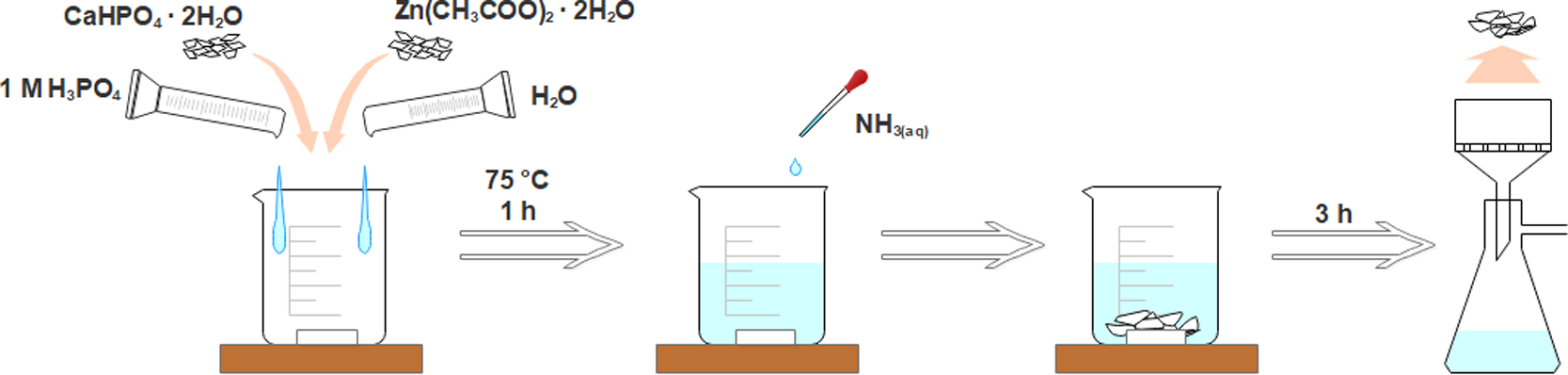
Schematic representation of the synthesis of Zn-WH powders by the dissolution–precipitation method.

For the synthesis of Zn-WH powders, calcium hydrogen phosphate dihydrate (CaHPO_4_·2H_2_O, 99.1%, Eurochemicals) and zinc acetate dihydrate [Zn(CH_3_COO)_2_·2H_2_O, ≥99.5%, Roth] were used as starting materials. All chemicals were used as received without additional purification. To achieve variable chemical compositions of the products, metal-ion precursors were mixed in various proportions. In a typical synthesis, certain amounts of CaHPO_4_·2H_2_O and Zn(CH_3_COO)_2_ corresponding to Ca-to-Zn molar ratios of 9, 10, 12, 15, 20, and 30 were dissolved in a mixture of 100 mL of distilled water and 13 mL of 1 M phosphoric acid (H_3_PO_4_, 75%, Roth). The total concentration of metal ions in the reaction mixture was 0.065 M. The temperature of the obtained solution was set to 75 °C, and the mixture was stirred for 1 h. Next, under constant mixing on a magnetic stirrer, concentrated ammonia solution (NH_4_OH, 25%, Roth) was added in order to adjust the pH to 5.6. The increase of the pH value of the reaction medium resulted in instantaneous formation of white precipitates. The resulting mixture was stirred for 3 h at 75 °C; afterward the precipitates were vacuum-filtered, washed with distilled water, and dried at 60 °C in an oven overnight. Hereafter, the synthesized powders will be indicated in the text by initial Ca-to-Zn ratio in the reaction mixture. Schematic representation of the synthesis procedure is illustrated in Figure 1.

### 2.2 Characterization

Powder XRD data of synthesized specimens were obtained using a Rigaku MiniFlex II diffractometer (Cu Kα, λ = 1.5419 Å) working in the Bragg–Brentano (θ/2θ) geometry. The data were obtained within the 10–60° 2θ angle range with a speed of 1°/min. Fourier transform infrared spectra (FTIR) were recorded in the range of 4000–400 cm^–1^ with a Bruker ALPHA-FTIR spectrometer. Raman spectra were recorded using a combined Raman and scanning near-field optical microscope WiTec Alpha 300 R equipped with a 532 nm excitation laser source. Elemental composition of synthesized compounds was determined using inductively coupled plasma optical emission spectrometry (ICP–OES) with a PerkinElmer Optima 7000 DV spectrometer. The morphology of synthesized powders and elemental distribution were analyzed by scanning electron microscopy (SEM) using a Hitachi SU-9000 microscope equipped with an energy-dispersive X-ray spectrometer. Transmission electron microscopy (TEM) analysis was performed on a JEOL JEM-2100F FEG TEM instrument.

## 3 Results and Discussion

**Figure 2:**
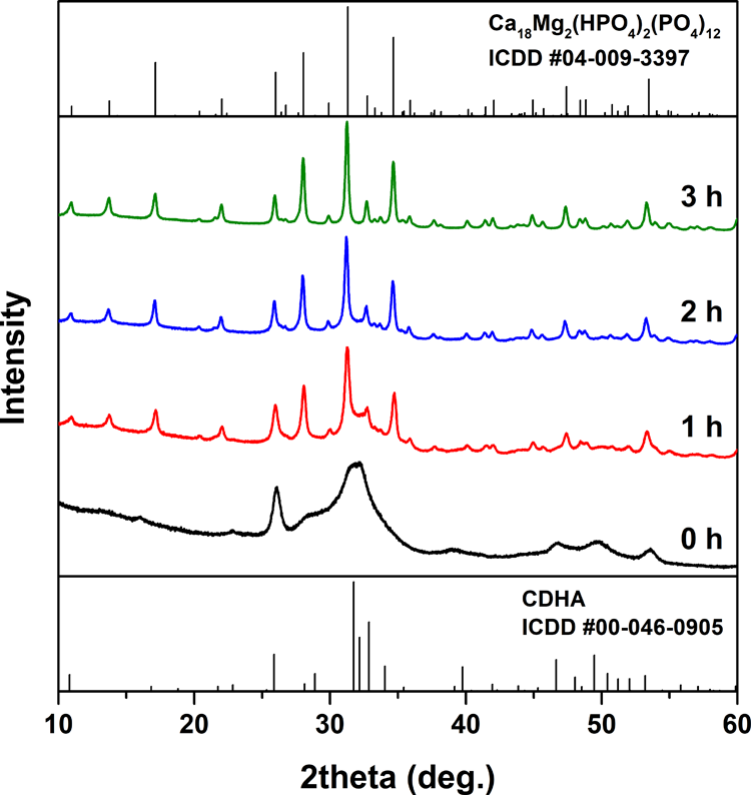
XRD patterns of synthesis products as a function of reaction time (the Ca-to-Zn ratio is 10).

The XRD patterns of the synthesis products as a function of reaction time are represented in Figure 2. It was observed that as-precipitated (0 h) powders possess a low-crystallinity calcium-deficient hydroxyapatite (CDHA) crystal structure (ICDD #00-046-0905); there were no diffraction peaks corresponding to WH, brushite (CaHPO_4_·2H_2_O), or any other crystalline material. With an increase of reaction time, gradual transformation of CDHA to WH occurred. A mixture of two phases was obtained when the reaction time was 1 and 2 h, while single-phase WH was observed after 3 h. As seen, all X-ray reflection peaks correspond to the WH crystal phase and peak positions match well with those of Mg-WH (ICDD #04-009-3397). The absence of reflections related to phases other than WH indicates high phase purity of the powders. Based on these results, the reaction time of 3 h was assumed to be optimal and all further syntheses were carried out for 3 h. Such a quick conversion of CDHA to WH was surprising when taking into account previous studies on the preparation of Mg-WH. Jang et al.^9^ synthesized Mg-WH powders by the dissolution–precipitation method through the conversion of CDHA to Mg-WH in the presence of Mg ions. Phase-pure Mg-WH was obtained only after 12 h of reaction at 80 °C or 24 h at 65 °C, which is significantly longer compared to our results. Moreover, in our case, we did not observe any intermediate CP phase, such as brushite. Instead, phase conversion occurred directly from CDHA to WH. In a separate study by Wang et al.,^7^ the preparation of Mg-WH required hydrothermal conditions at 200 °C for a 12 h treatment, which is also a considerably long time. In this light, our proposed method is time-efficient, which is a very beneficial synthetic feature.

**Figure 3:**
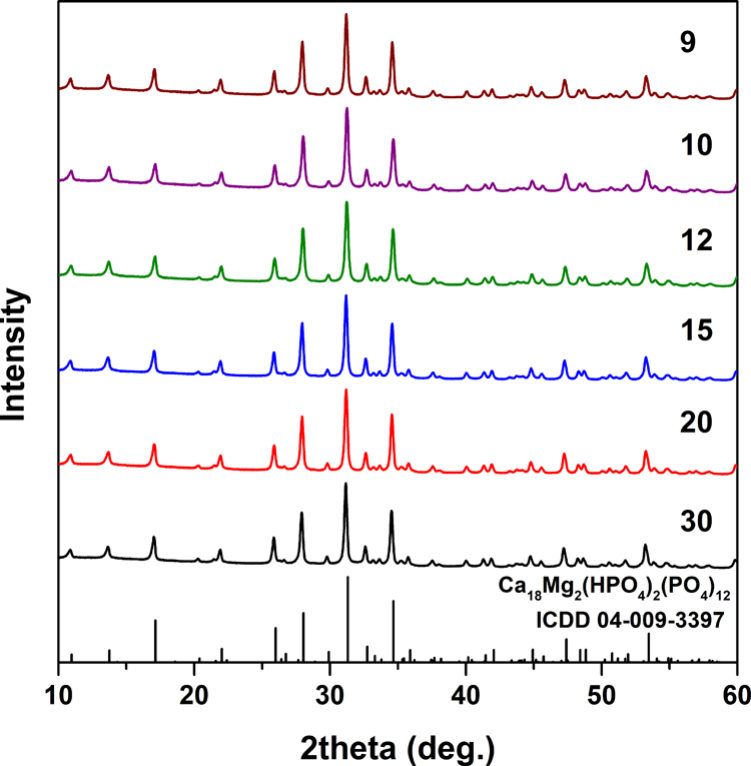
XRD patterns of Zn-WH powders synthesized with different Ca-to-Zn ratios.

The XRD patterns of Zn-WH synthesized with different Ca-to-Zn ratios in the reaction mixture are illustrated in Figure 3. Evidently, single-phase Zn-WH powders without a trace of crystalline byproducts were successfully synthesized when the Ca-to-Zn ratio in the reaction medium was in the range from 9 to 30. With higher Zn amounts, a secondary phase, scholzite [CaZn_2_(PO_4_)_2·_2(H_2_O)] was formed, whereas with lower amounts of Zn, a mixture of WH and CDHA was obtained (see Figure S1). These results demonstrate that a WH structure can be formed when the initial ratio of metal ions is different compared to stoichiometric WH [Ca_18_M_2_(HPO_4_)_2_(PO_4_)_12_]. The obtained results supplement the data reported on the synthesis of Mg-WH.^9^ Previously reported synthesis conditions and the suggested phase diagram deduced that the formation of Mg-WH occurred with an excess of Mg and phosphate ions. In our case, we also have an excess of phosphates; however, Zn-WH compounds were obtained with Zn amounts lower than those in the nominal WH formula. Moreover, as mentioned, we were not able to prepare single-phase Zn-WH from the Zn-rich reaction mixture, when the Ca-to-Zn ratio was lower than 9.

**Figure 4:**
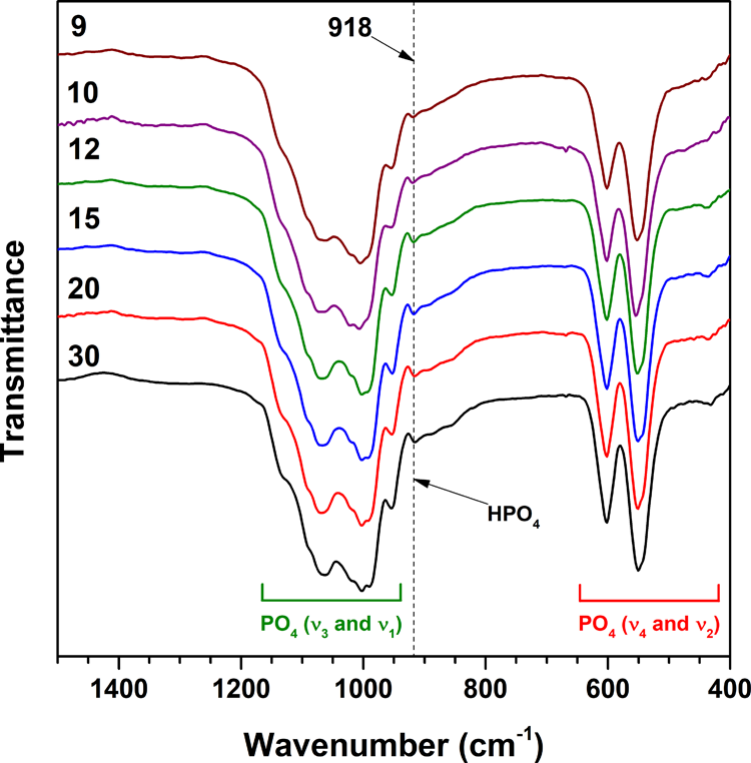
FTIR spectra of Zn-WH powders synthesized with different Ca-to-Zn ratios.

As previously mentioned, it is hard to distinguish the XRD patterns of WH and β-TCP; therefore, the use of vibrational spectroscopy is crucial for the full characterization of WH powders and confirmation of the presence of distinct functional groups. Infrared and Raman spectroscopies use chemical functional group frequency analysis to identify the molecular components of the substances. These techniques are sensitive to the crystallographic site symmetry of the material, which allows distinction among crystallographically similar structures. The FTIR spectra of synthesized Zn-WH powders in the representative spectral range of 1500–400 cm^–1^ are demonstrated in Figure 4.

The most intense absorption bands correspond to the vibrations of phosphate functional groups. The absorption bands in the range from approximately 1200 to 930 cm^–1^ correspond to the phosphate ν_3_ and ν_1_ stretching modes. The bands in the 640–500 cm^–1^ region and at 436 cm^–1^ are also characteristic of phosphate groups and ascribed to the ν_4_ and ν_2_ bending modes, respectively.^29^ The shape of these bands is nearly the same for all compounds regardless of the amounts of Ca and Zn precursors used for the synthesis. For the identification of WH, attention must be paid to the absorption bands located at 918 and 865 cm^–1^, indicative of HPO_4_^2–^.^7,8,30^ While the band at 865 cm^–1^ is not so pronounced, the band at 918 cm^–1^ is clearly visible in all presented spectra, suggesting a WH phase. Moreover, it should be noted that the relative intensity of this band is comparable for all compounds, which suggests that the number of HPO_4_^2–^ groups does not depend significantly on the content of smaller Zn cations. Both cationic and anionic substitutions in phosphates can cause changes in the FTIR spectra such as broadening and shifting of the position of absorption bands.^31,32^ For instance, Bigi et al.^33^ demonstrated that partial substitution of Ca^2+^ with Zn^2+^ in β-TCP leads to the degeneracy of PO_4_^3–^ absorption bands. In our case, we did not observe any drastic changes correlated to the amounts of Ca and Zn precursors used for the synthesis; however, some subtle changes could be observed. For example, with increasing Zn content, the signals ascribed to the ν_4_ mode (640–500 cm^–1^) became more overlapped. The enlarged view of the FTIR spectra (Figure S2) demonstrates a negligible difference in the position of bands ascribed to HPO_4_^2–^ groups (ca. 918 cm^–1^).

**Figure 5:**
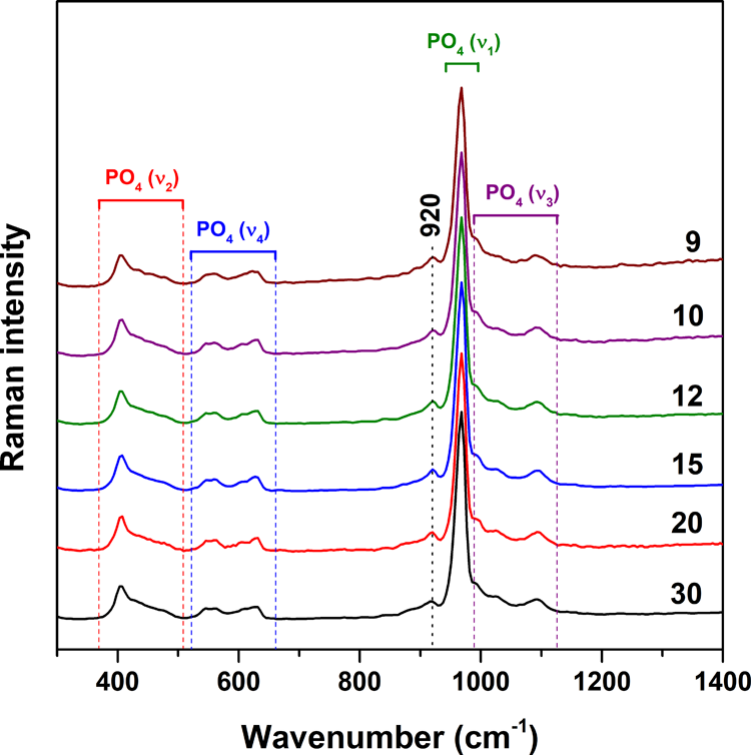
Raman spectra of Zn-WH powders synthesized with different Ca-to-Zn ratios.

The room-temperature Raman spectra of synthesized Zn-WH specimens are given in Figure 5. Characteristic bands can be observed in the ranges of approximately 370–510 (ν_2_), 530–645 (ν_4_), and 990–1125 cm^–1^ (ν_3_). The most intense band centered at 965 cm^–1^ is ascribed to the ν_1_ symmetric-stretching vibrational mode. All these bands are also present in the Raman spectra of β-TCP and associated with internal vibrations of PO_4_^3–^ ions.^34^ The remarkable feature of all obtained Raman spectra is the clearly visible band at 920 cm^–1^, which is the characteristic spectral marker for HPO_4_^2–^.^35,36^ This band is absent in the Raman spectrum of β-TCP.^29,37^ Another obvious difference compared to β-TCP is that the signal corresponding to the ν_1_ mode in the Raman spectrum of β-TCP is usually observed as a doublet;^29,34^ however, in our case, we can see only a single peak. Possibly, this spectral change can be caused by the low crystallinity of our prepared WH powders since the synthesis was performed at low temperature. A previously reported Raman spectrum of terrestrial WH exhibited a strong and well-resolved doublet of the ν_1_ band.^35^ On the other hand, in ion-substituted β-TCP, this band can also be observed as a singlet.^37^ Similarly, like in the FTIR spectra, there is no significant difference in relative intensity of observed bands depending on the chemical composition of the samples; particularly, the relative intensity of the band at 920 cm^–1^ does not change depending on the Ca-to-Zn ratio. This observation suggests that the number of HPO_4_^2–^ groups in all synthesized specimens is equal or very similar. A closer look (Figure S3) shows that the position of the ν_1_ band does not change in the series. A very negligible difference in the position of the HPO_4_^2–^-related signal (ca. 920 cm^–1^) can be observed as in the case of the FTIR spectra. Overall, it can be concluded that vibrational spectroscopy supported the results obtained by XRD analysis (Figure 2) and confirmed the WH structure of the compounds.

In order to check the chemical composition of synthesized products, the elemental analysis by means of ICP–OES was performed. The results are summarized in Table 1. It is evident that with an increase of Zn concentration in the reaction mixture, Zn content in synthesized powders increased as well. The determined Ca-to-Zn ratio in the products is very close to the initial ratio of metals introduced in the reaction mixture. At the same time, the total metal ions to P ratio regardless of starting ratios of metal ions was maintained close to 1.428, which is the ratio in the ideal WH with the formula Ca_18_Zn_2_(HPO_4_)_2_(PO_4_)_12_. These results, together with the data of XRD, FTIR, and Raman spectroscopies, demonstrate that the crystal structure of WH can be formed with lower contents of smaller cations and when the Ca-to-Zn ratio exceeds the nominal value of 9. To our knowledge, the phenomenon of the formation of small-cation-lean WH (Zn-lean in our case) is a very new one, which was not described previously. While the only existing system for comparison is Mg-WH, it is not surprising that Mg-lean WH was not reported since it is not an expected product of the synthesis procedure in a Mg-rich medium.^7−9,13^ These observations open new horizons for the structural investigations of WH-type materials.

**Table 1:** Results of the Elemental Analysis of Zn-WH Powders

initial Ca-to-Zn molar ratio	actual Ca-to-Zn molar ratio	actual M-to-P molar ratio
30	29.7	1.41
20	18.5	1.41
15	14.5	1.40
12	12.2	1.41
10	9.85	1.40
9	8.94	1.41

**Figure 6:**
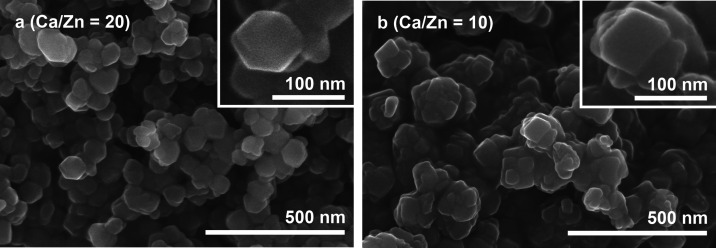
SEM micrographs of Zn-WH powders synthesized with Ca-to-Zn ratios of 20 (a) and 10 (b).

The representative SEM micrographs of Zn-WH powders synthesized with Ca-to-Zn ratios of 20 and 10 are shown in Figure 6. It is seen that powders synthesized with a Ca-to-Zn ratio of 20 (Figure 6a) consist of mostly uniform and agglomerated particles. The size of individual particles varies in the range of approximately 60–80 nm. Despite the fact that synthesized powders were highly agglomerated, a closer look shows that some particles have a very distinctive hexagonal shape (inset of Figure 6a). An even higher degree of agglomeration was observed for the Zn-WH sample synthesized with a higher Zn amount (Figure 6b) as the obtained particles were closely stacked on each other. It is interesting to note that the shape of the particles was found to be dependent on the chemical composition of the powders. With an increase of Zn content, the shape evolution of Zn-WH crystals from hexagonal to rhombohedral was observed. The hexagonal shape was previously achieved for Mg-WH and β-TCP powders synthesized by different methods.^7,10,38−42^ Guo et al.^38^ demonstrated that the shape of the grains of Mg-WH grown on the surface of β-TCP pellets under hydrothermal conditions can be varied by changing the reaction time. Wang et al.^7^ achieved morphology control of Mg-WH powders by varying the ratio of Mg and Ca precursors. Mg-WH crystals with a rhombohedral shape were previously obtained by Jang et al.^9^ The SEM micrographs of Zn-WH powders synthesized with other Ca-to-Zn ratios as well as images taken at lower magnification are given in Figures S4 and S5. It is shown that there are no crystals with obviously different morphologies, which can be considered as additional indirect evidence of the phase purity of the products.

**Figure 7:**
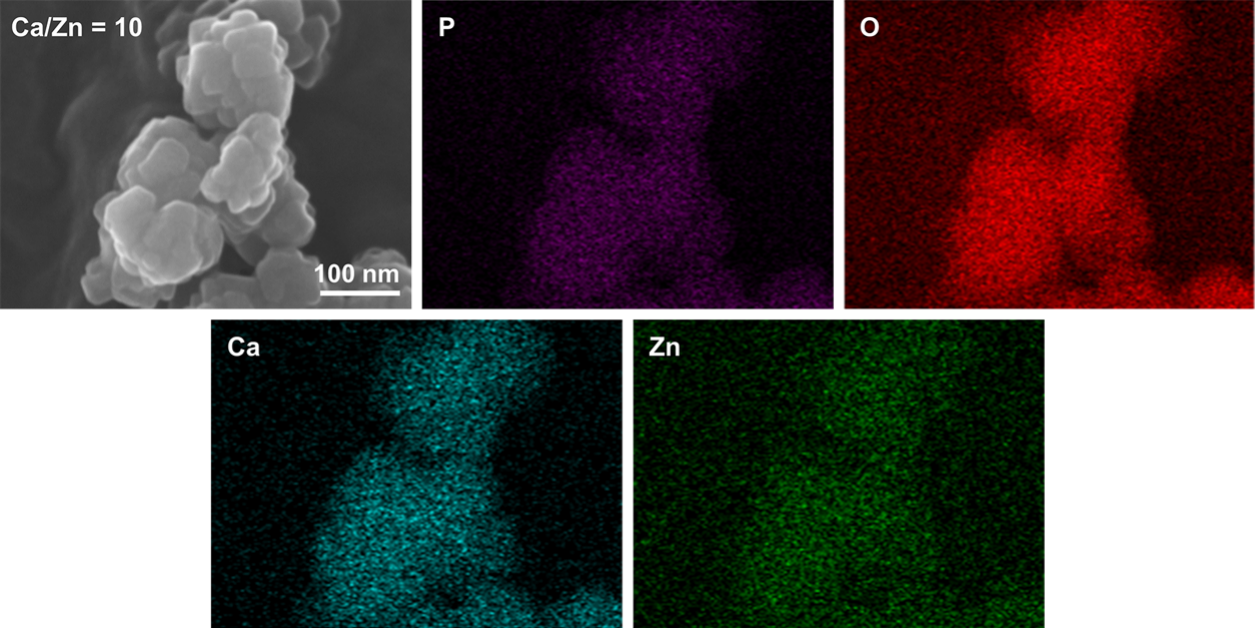
SEM micrograph and EDX mapping of Zn-WH powders synthesized with a Ca-to-Zn ratio of 10.

Figure 7 demonstrates the SEM image and EDX mapping of Zn-WH samples. These results confirm uniform distribution of all elements in Zn-WH powders; there are no visible regions with high concentrations of some elements and complete absence of others. This indicates an absence of neighboring Zn-rich phases, which might not be detected by XRD or vibrational spectroscopy.

**Figure 8:**
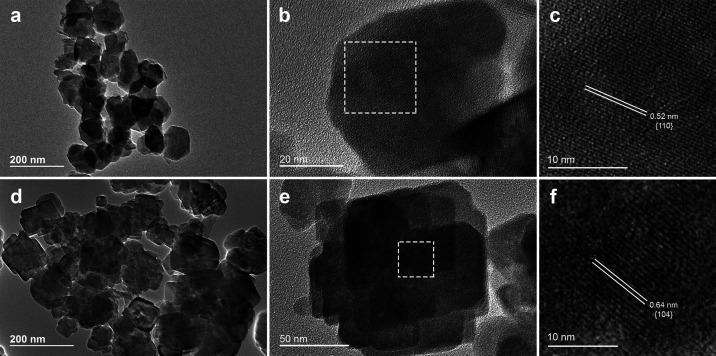
TEM images of Zn-WH synthesized with Ca-to-Zn ratios of 20 (a–c) and 10 (d–f).

TEM images of Zn-WH powders are depicted in Figure 8. These images agree with the results obtained by SEM and confirm the presence of hexagonal plates with clearly defined sides in the Zn-WH sample synthesized with a Ca-to-Zn ratio of 20 (Figure 8a). High-resolution TEM of a single-crystalline hexagonal plate revealed a d-spacing of 0.52 nm, which is the characteristic of (110) lattice planes. The top/bottom surface of the plate was identical to (001) facets of WH, which is in good agreement with previous studies.^7,41,42^ Well-defined rhombohedral particles of Zn-WH (a Ca-to-Zn ratio of 10) can be seen in Figures 8d,e. Fast Fourier transform of the top crystal revealed a d-spacing of 0.64 nm, which corresponds to the (104) lattice planes; in this case, the top/bottom surface of the plate was assigned to (10–2) facets. This observed structural geometry also coincides well with that reported for Mg-WH.^15^

**Figure 9:**
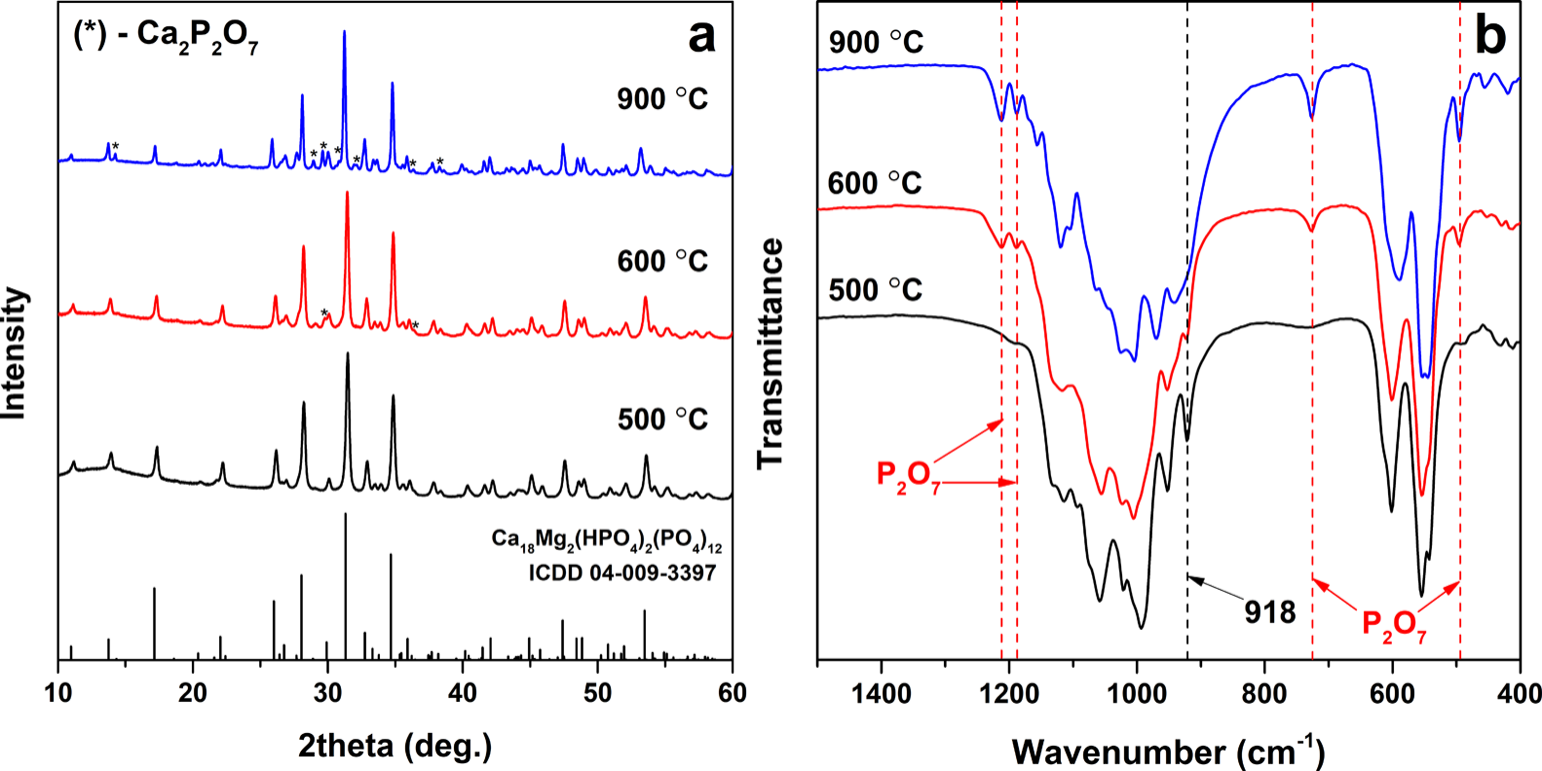
XRD patterns (a) and FTIR spectra (b) of Zn-WH powders annealed at different temperatures (the Ca-to-Zn ratio is 10).

In order to check thermal stability and to estimate a potential use of the synthesized Zn-WH powders for the high-temperature fabrication of ceramics, the samples were annealed at different temperatures. The XRD patterns and FTIR spectra of Zn-WH powders annealed at different temperatures are depicted in Figure 9. No visible changes and newly aroused diffraction peaks were noticed in the XRD pattern after annealing at 500 °C; however, after the heat treatment at higher temperatures, the appearance of additional peaks was observed (Figure 9a). The newly formed crystalline phase was identified as Ca_2_P_2_O_7_ (PDF #00-081-2257). A similar trend can be seen in the FTIR spectra (Figure 9b). It should be noted that the absorption band at 918 cm^–1^, corresponding to HPO_4_^2–^, after annealing at 500 °C became more intense compared to the FTIR spectra of the as-synthesized powders (Figure 4). A possible explanation of this change could be found in the increase of degree of crystallinity after the heat treatment. After annealing at higher temperatures, this band gradually disappeared, while at the same time, new absorption signals arose at 495, 726, 1187, and 1211 cm^–1^. These bands confirm the formation of Ca_2_P_2_O_7_.^43^ Making an assumption that all Zn ions are transferred to the β-TCP structure, the water release and degradation of Zn-WH can be described by the following reaction:
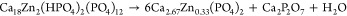
1

The same behavior was observed for all Zn-WH powders synthesized with other Ca-to-Zn ratios; moreover, the relative intensity of the diffraction peaks corresponding to Ca_2_P_2_O_7_ did not depend on the chemical composition of initial compounds (see Figure S6). According to Gopal and Calvo,^44^ Mg-WH transforms to Mg-doped β-TCP at around 900 °C; however, the authors do not mention about the formation of a secondary crystalline phase. On the other hand, Jang et al.^8^ did not observe any appearing crystal structure or changes in the XRD pattern of Mg-WH after heat treatment to temperatures of up to 1450 °C. In our case, it is evident that the intensity of both diffraction peaks and absorption bands corresponding to Ca_2_P_2_O_7_ increases as annealing temperature increases, indicating the thermally unstable nature of the synthesized materials.

Summarizing the obtained results, it can be concluded that the new material for the potential use in regenerative medicine was synthesized. Further studies will be focused on the behavior of Zn-WH under physiological conditions and rigorous investigation of structural and biological properties.

## 4 Conclusions

Zinc whitlockite powders with variable metal content have been synthesized by a low-temperature dissolution–precipitation process. Complete phase transformation from CDHA to zinc whitlockite occurred in an acidic medium in the presence of Zn^2+^ ions. Controllable chemical composition of the synthesis products was achieved by changing the initial Ca-to-Zn molar ratio in the reaction mixture. Regardless of the final Ca-to-Zn ratio in the obtained products, the total metal ions to phosphorus ratio was determined to be nearly constant, indicating the formation of a whitlockite structure with stoichiometric and Zn-lean composition. The morphology of the powders can be controlled by varying the metal-ion ratio in the reaction mixture. All synthesized compounds were determined to be thermally unstable and decomposed upon heat treatment with the formation of β-TCP and Ca_2_P_2_O_7_.

## Supporting Information Available

XRD patterns of the products synthesized with other Ca-to-Zn ratios; enlarged view of FTIR and Raman spectra; additional SEM images of Zn-WH powders; and XRD patterns of annealed Zn-WH powders (PDF)
