# Supplementary material for: Dissolution–Precipitation Synthesis and Characterization of Zinc Whitlockite with Variable Metal Content
Source: ACS Biomater Sci Eng. 2021 Jul 28;7(8):3586–93. doi: 10.1021/acsbiomaterials.1c00335 (PMC8396804; doi:10.1021/acsbiomaterials.1c00335)
Supplement: Supplementary file 1 — ab1c00335_si_001.pdf [file ab1c00335_si_001.pdf]

Supporting information

Dissolution-Precipitation Synthesis and Characterization of Zinc Whitlockite with Variable Metal Content

Agne Kizalaite[1], Inga Grigoraviciute-Puroniene[1], Dane Romar C. Asuigui[2], Sarah L. Stoll[2], Sung Hun Cho[3], Tohru Sekino[3], Aivaras Kareiva[1], Aleksej Zarkov[1,*]

[1]*Institute of Chemistry, Vilnius University, Naugarduko 24, LT-03225 Vilnius, Lithuania*

[2]*Department of Chemistry, Georgetown University, 37th and O Streets NW, Washington, D.C. 20057, United States*

[3]*SANKEN (The Institute of Scientific and Industrial Research), Osaka University, 8–1 Mihogaoka, Ibaraki, Osaka 567–0047, Japan*

**Corresponding author: E-mail: aleksej.zarkov@chf.vu.lt;*

Page S2. Figure S1. XRD patterns of Zn-WH synthesized with different Ca-to-Zn ratios (7.5, 8.5 and 60).

Page S3. Figure S2. Enlarged view of the FTIR spectra of Zn-WH synthesized with different Ca-to-Zn ratios.

Page S4. Figure S3. Enlarged view of the Raman spectra of Zn-WH synthesized with different Ca-to-Zn ratios.

Page S5. Figure S4. SEM micrographs of Zn-WH powders synthesized with Ca-to-Zn ratios 30 (a) and 9 (b).

Page S6. Figure S5. Low magnification SEM micrographs of Zn-WH powders synthesized with Ca-to-Zn ratios 30 (a), 20 (b), 10 (c) and 9 (d).

Page S7. Figure S6. XRD patterns of Zn-WH powders synthesized with different Ca-to-Zn ratios and annealed at 900 ºC.

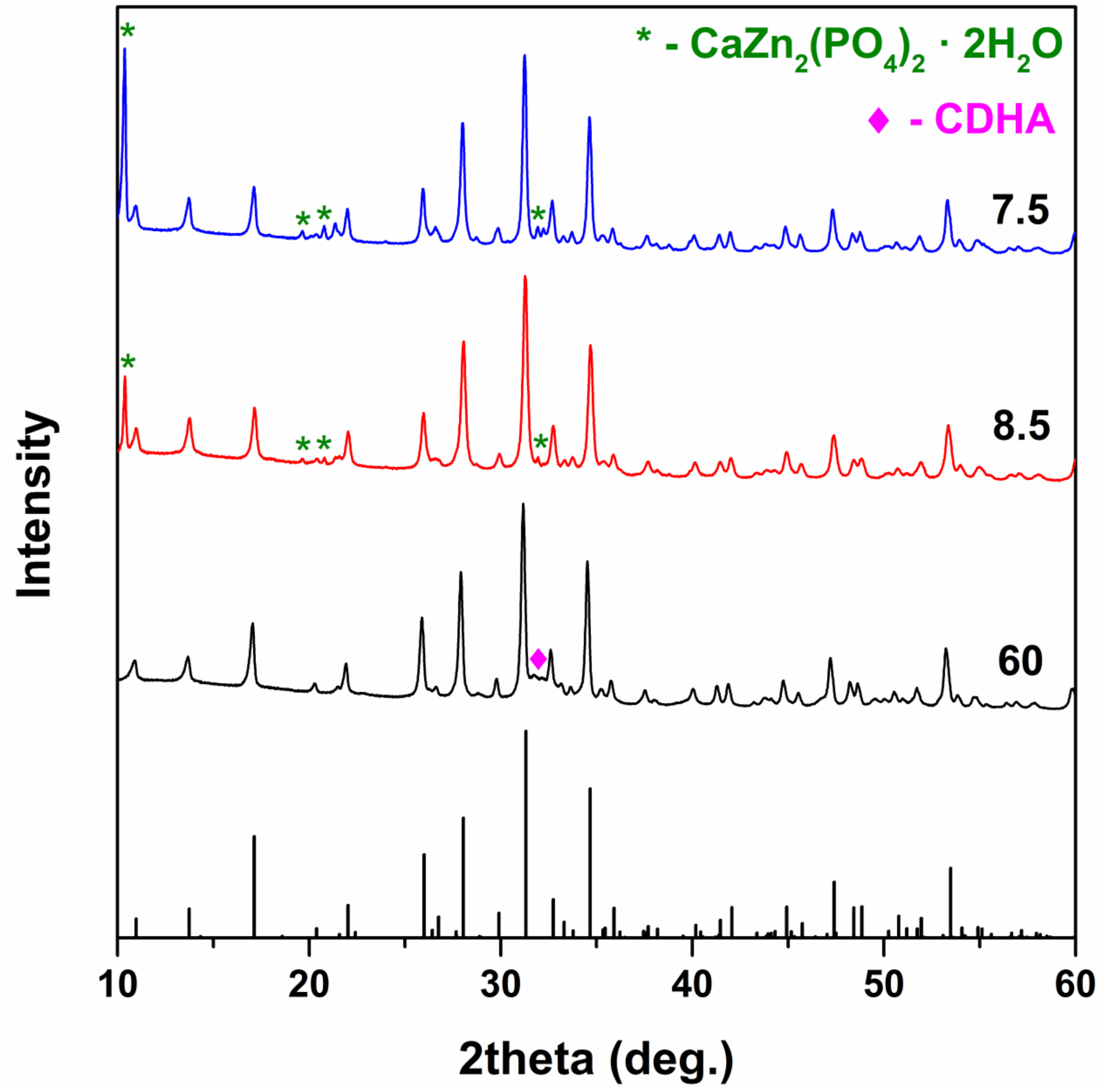

Figure S1. XRD patterns of Zn-WH synthesized with different Ca-to-Zn ratios (7.5, 8.5 and 60).

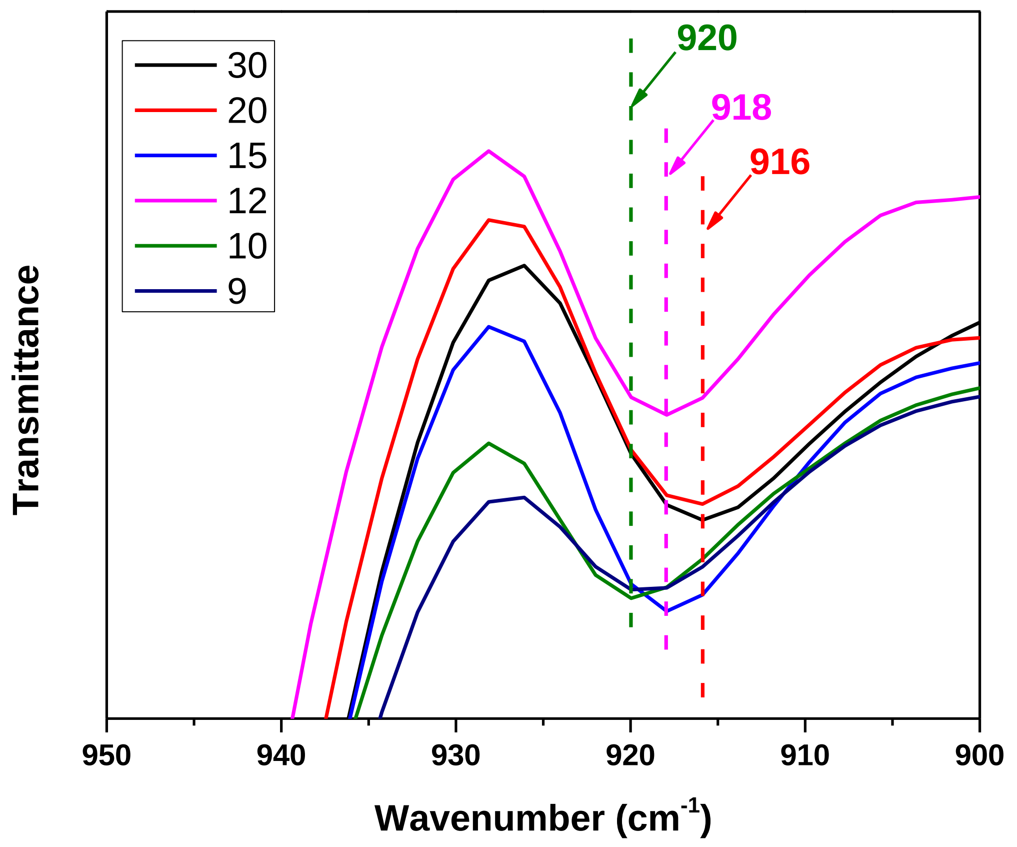

Figure S2. Enlarged view of the FTIR spectra of Zn-WH synthesized with different Ca-to-Zn ratios.

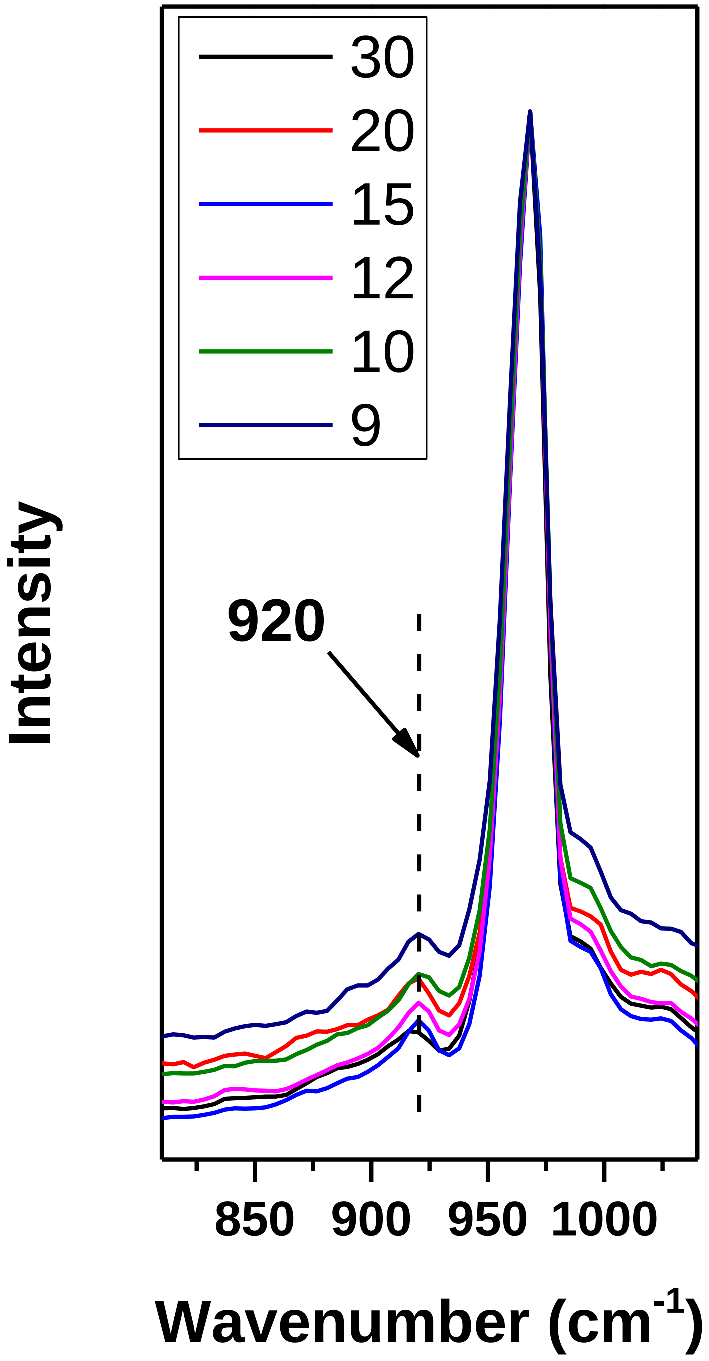

Figure S3. Enlarged view of the Raman spectra of Zn-WH synthesized with different Ca-to-Zn ratios.

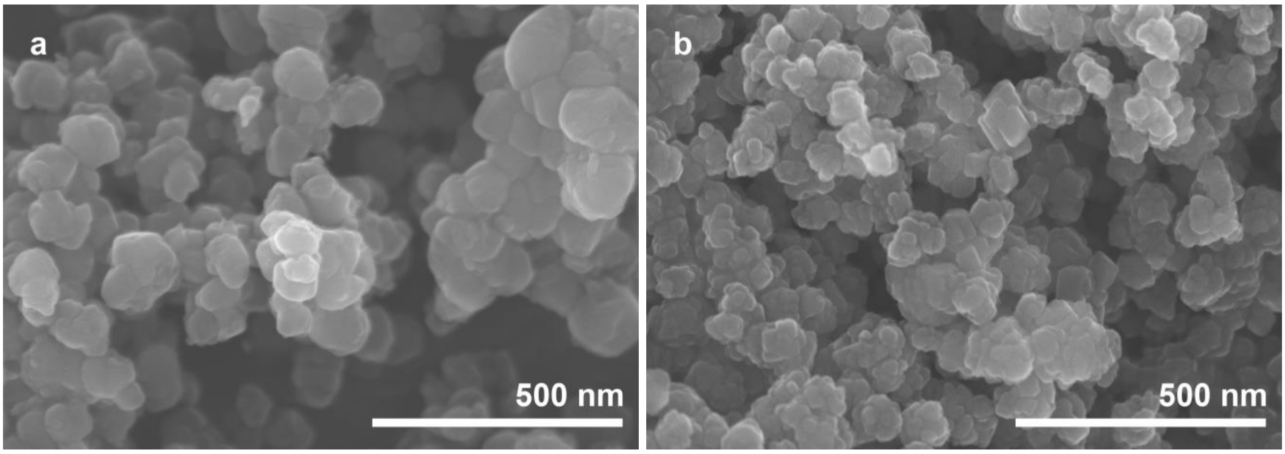

Figure S4. SEM micrographs of Zn-WH powders synthesized with Ca-to-Zn ratios 30 (a) and 9 (b).

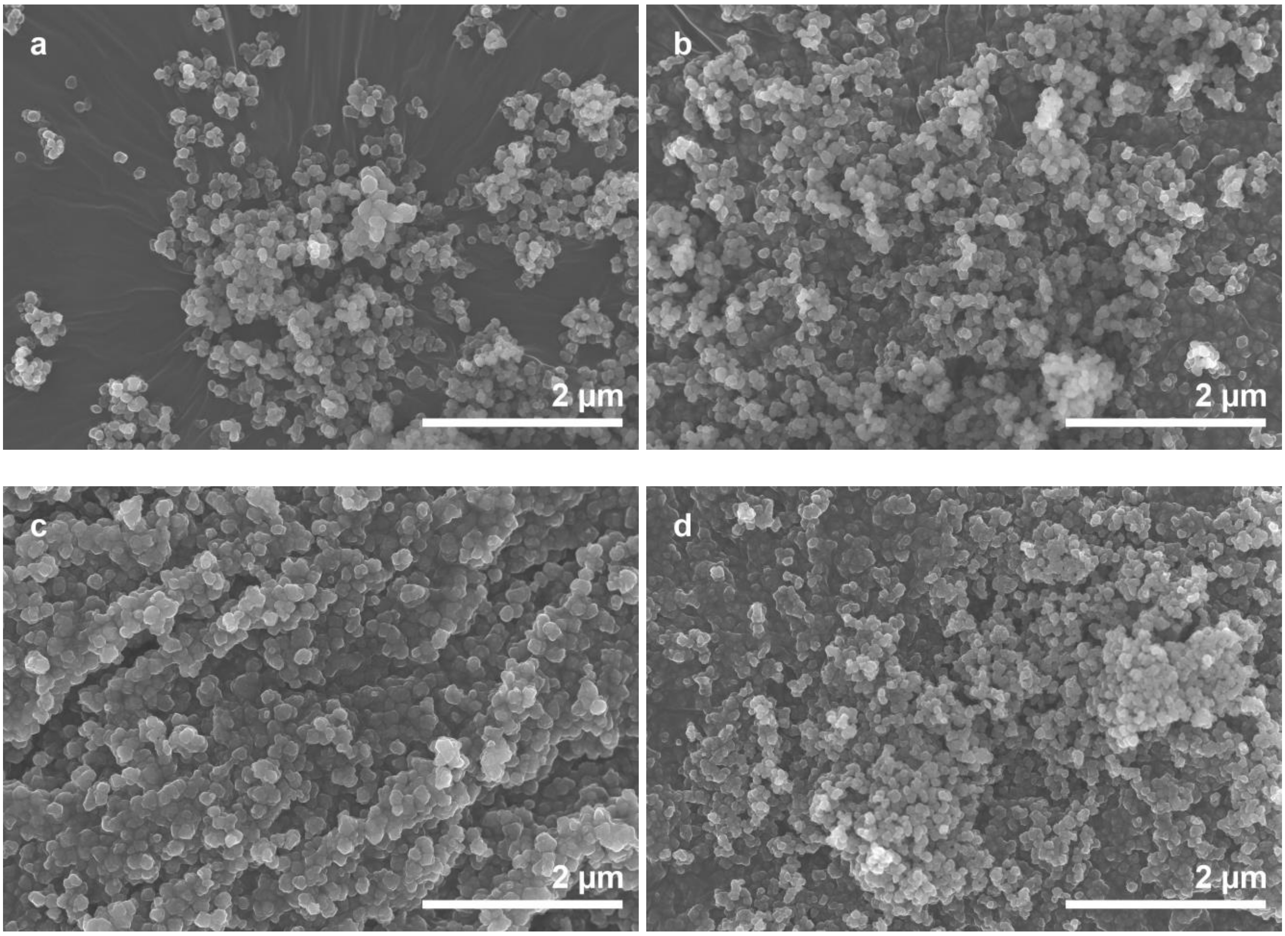

Figure S5. Low magnification SEM micrographs of Zn-WH powders synthesized with Ca-to-Zn ratios 30 (a), 20 (b), 10 (c) and 9 (d).

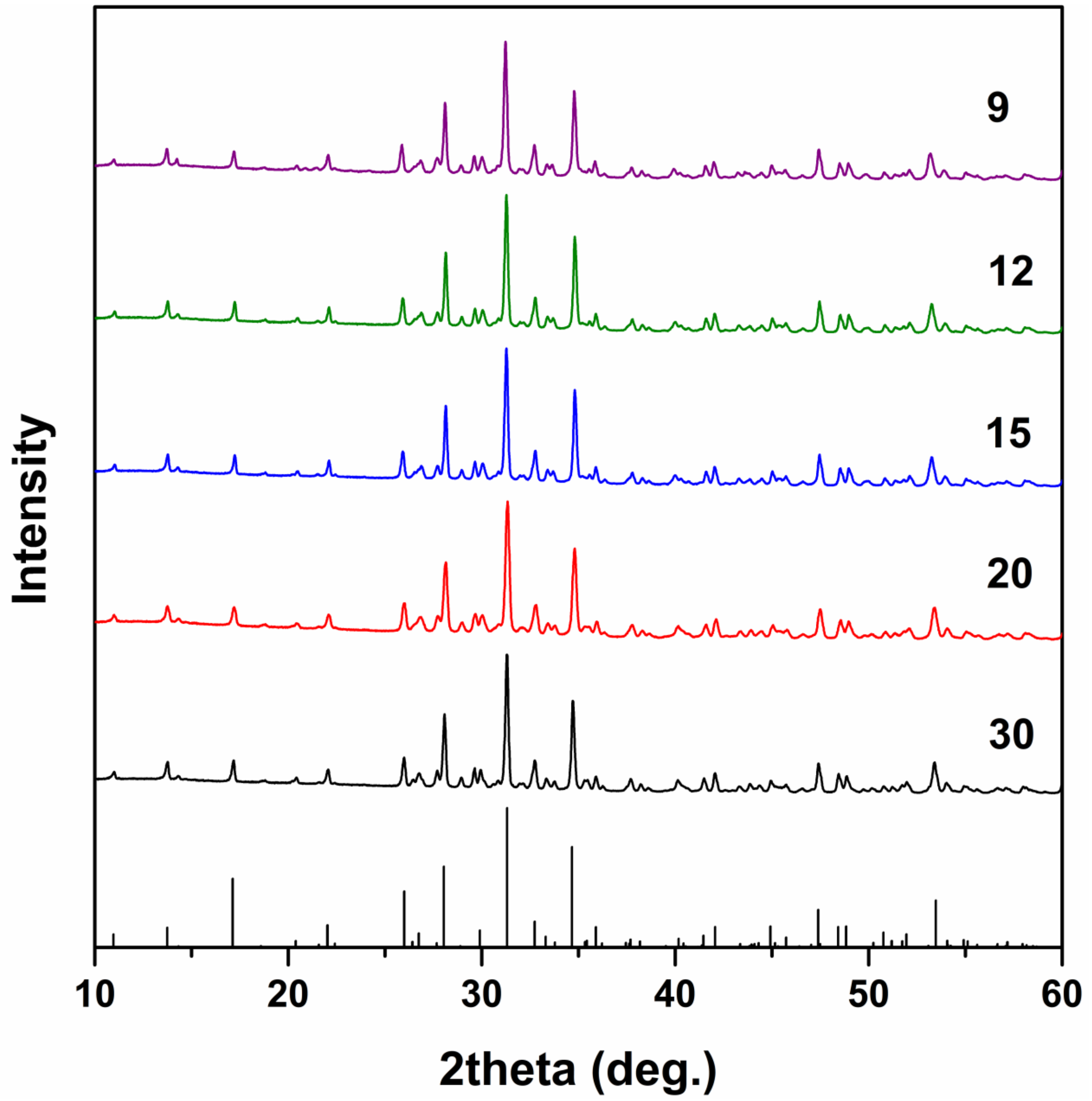

Figure S6. XRD patterns of Zn-WH powders synthesized with different Ca-to-Zn ratios and annealed at 900 ºC.
